# Analysis of the Influence of Both the Average Molecular Weight and the Content of Crosslinking Agent on Physicochemical Properties of PVP-Based Hydrogels Developed as Innovative Dressings

**DOI:** 10.3390/ijms231911618

**Published:** 2022-10-01

**Authors:** Magdalena Kędzierska, Mateusz Jamroży, Anna Drabczyk, Sonia Kudłacik-Kramarczyk, Magdalena Bańkosz, Mateusz Gruca, Piotr Potemski, Bożena Tyliszczak

**Affiliations:** 1 Department of Chemotherapy, Medical University of Lodz, Copernicus Memorial Hospital of Lodz, 93-513 Lodz, Poland; 2 Faculty of Materials Engineering and Physics, Cracow University of Technology, 37 Jana Pawła II Av., 31-864 Krakow, Poland; 3 Department of Materials Engineering, Faculty of Materials Engineering and Physics, Cracow University of Technology, 37 Jana Pawła II Av., 31-864 Krakow, Poland

**Keywords:** hydrogels, L-ascorbic acid, *Aloe vera*, crosslinking density, crosslinking agent, tensile strength, elongation, surface morphology, swelling, SEM imaging

## Abstract

Hydrogels belong to the group of polymers with a three-dimensional crosslinked structure, and their crosslinking density strongly affects their physicochemical properties. Here, we verified the impact of both the average molecular weight of crosslinking agents used during the photopolymerization of hydrogels and that of their content on selected properties of these materials. First, PVP-based hydrogels modified with *Aloe vera* juice and L-ascorbic acid were prepared using UV radiation. Next, their surface morphology was characterized via optical scanning electron microscopy, whereas their chemical structure was investigated by FT-IR spectroscopy. Moreover, we verified the tendency of the hydrogels to degrade in selected physiological liquids, as well as their tensile strength, percentage of elongation, and swelling capability. We found that the more crosslinking agent in the hydrogel matrix, the higher its tensile strength and the less elongation. The hydrogels showed the highest stability during incubation in SBF and 2% hemoglobin solution. A sharp decrease in the pH of distilled water observed during the incubation of the hydrogels was probably due to the release of *Aloe vera* juice from the hydrogel matrices. This was additionally confirmed by the decrease in the intensity of the absorption band derived from the polysaccharides included in this additive and by the decrease in the swelling ratio after 48 h. Importantly, all hydrogels demonstrated swelling properties, and it was proven that the higher content of the crosslinking agent in hydrogels, the lower their swelling ability.

## 1. Introduction

Polyvinylpyrrolidone (PVP) belongs to the group of synthetic polymers and is increasingly used to develop various biomaterials, owing to its interesting properties, including hemo- and biocompatibility, low cytotoxicity, affinity to a wide variety of both hydrophilic and hydrophobic substances, and biodegradability. Importantly, PVP also shows promising properties in terms of its processability, i.e., very good solubility both in various organic solvents and in water, as well as high chemical and thermal resistance [1,2,3]. Therefore PVP constitutes a component of many materials designed for applications in tissue engineering [4,5,6] and controlled drug delivery systems [7,8,9,10] or as innovative dressing materials [11,12,13].

In particular, there has been a recent trend of increasing interest in designing wound dressing materials using PVP. For example, Contardi et al. reported on PVP-based electrospun fibrous hydrogels incorporated with hydroxycinnamic acid derivatives, proving that such dressings showed both hemo- and biocompatibility, as well as antioxidant activity, and, importantly, promoted skin regeneration [14]. Fibrous dressings based on PVP were also described by Sun et al., who applied silk fibroin and PVP as base components of nanofibers, using phlorizin as a modifier. Investigations showed that the PVP-containing nanofibers were characterized by both antioxidant and antibacterial activity, as well as promising mechanical properties and hydrophilicity. It was also demonstrated that these materials supported the wound regeneration processes [15]. PVP-based fibers investigated in terms of their usefulness as dressing materials were also discussed by Alipour et al. [16] and Aytimur and Uslu [17]. In other work, PVP, chitosan and silver oxide nanoparticles were used to fabricate dressing materials in the form of films. Such designed dressings demonstrated good antibacterial properties, swelling ability, and transparency (important in terms of the possibility of constant wound condition monitoring) [18]. Poonguzhali et al. proposed bionanocomposite membranes based on PVP and chitosan additionally reinforced with nanocellulose as innovative dressings. The materials were prepared by means of the salt leaching method and exhibited antibacterial activity. Importantly, they accelerated the wound healing process by supporting wound contraction and re-epithelization [19]. In another study, PVP, chitosan, and titanium dioxide were applied by Archana et al. to develop nanocomposite dressings. During the experiments, it was proven that the nanocomposites were characterized by high sorption capacity, hydrophilicity, and biocompatibility toward both L929 and NH3T3 fibroblast cell lines. Importantly, the developed dressings supported healing of open excision-type wounds, which was demonstrated during in vivo experiments involving an albino rat model [20]. In other studies, researchers characterize PVP-containing dressings obtained as a result of polymerization radiation. For example, such a solution was proposed by Singh and Pal., who reported a high swelling capability of the developed materials, in addition their ability to release an active substance (doxycycline hyclate) in simulated wound fluid [21]. In turn, hydrogel dressings based on PVP and carboxymethyl cellulose crosslinked via ^60^Co γ-ray irradiation were investigated by Wang et al., who proved that compared to commercial dressings, the developed materials showed better swelling capability and a similar ability to retain moisture [22]. PVP-containing materials considered as wound dressings were also developed by de Lima et al. [23], Zhang et al. [24], and Varsei et al. [25].

In this paper, investigated the development of PVP-based hydrogel dressings synthesized via a photopolymerization process. The developed hydrogels were additionally modified with active substances (including *Aloe vera* juice and L-ascorbic acid (vitamin C)). The materials were then characterized in detail, with a focus on the impact of both the average molecular weight of the crosslinking agent used during hydrogel synthesis and the amount of reagent in the reaction mixture on the physicochemical properties of the hydrogels. According to literature reports, the crosslinking degree has an important impact on many properties of hydrogels [26,27]. For example, Ninciuleanu et al. proved that as the concentration of crosslinking agent increased, the water absorption ability of the tested hydrogels decreased [28]. Furthermore, Chavda et al. demonstrated that as the crosslinker concentration increased, the porosity and swelling ability of the tested hydrogels decreased [29]. A similar conclusion concerning the impact of crosslinking agent content in hydrogels on their swelling capacity was reported by Zhang et al, who also proved that as the crosslinker content increased, the elongation at break of the hydrogels decreased [30]. Additionally, Kopac et al. reported that the higher crosslinker concentration, the more homogenous the hydrogel [31]. Thus, we decided to use diacrylate poly(ethylene glycol) as a crosslinking agent and verify the influence of its average molecular weight (two types of this reagent were used: with an average molecular weight of 575 g/mol and 700 g/mol) and its content on selected hydrogel properties. We also evaluated the properties of the hydrogels in terms of their potential use as dressings. We investigated characteristics such swelling ability, surface morphology, and mechanical properties (tensile strength and percentage elongation). It was also important to determine whether the developed materials degrade in simulated physiological liquids.

## 2. Results and Discussion

### 2.1. Studies on the Tensile Strength and Percentage Elongation of the Hydrogels

**Figure 1:**
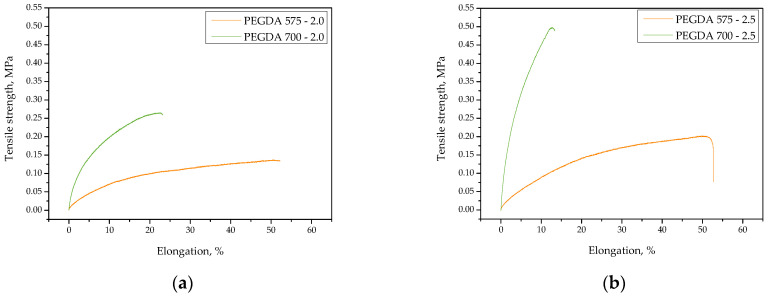
Stress–strains curves of hydrogels obtained using 2.0 mL (**a**) and 2.5 mL (**b**) of crosslinking agent with various average molecular weights.

**Figure 2:**
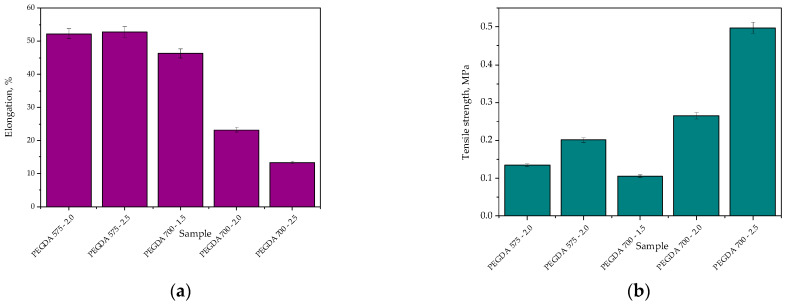
Percentage elongation of hydrogels (**a**) and their tensile strength (**b**).

Our first investigations involved determining the mechanical properties of hydrogels, i.e., their tensile strength and their percentage elongation. The obtained stress–strain curves of the tested samples are presented in Figure 1, whereas the values of the tensile strength and the percentage elongation of each hydrogel are summarized in bar charts shown in Figure 2.

The obtained results show that both the amount of the crosslinking agent used during the photopolymerization process and its average molecular weight affected both tensile strength and the percentage elongation of the hydrogels. It was not possible to perform mechanical investigations using sample PEGDA 575—1.5 (i.e., the sample obtained using the lowest amount of PEGDA (575)) due to its physicochemical properties, which made it impossible to fix this sample between the jaws of the testing machine.

We found that the higher the content of crosslinking agent in the hydrogel matrix, the higher its tensile strength. Increasing the amount of the reagent by 0.5 mL approximately doubled the sample’s tensile strength. This phenomenon might be explained by the crosslinking density of the hydrogel, which was strongly influenced by the concentration of crosslinking agent in the reaction mixture. An increase in reagent concentration results in increased crosslinking within the hydrogel network, as similarly reported by Wong et al. [32].

Importantly, the hydrogels synthesized using PEGDA 575 showed approximately half the tensile strength of those obtained using PEGDA 700. The use of a crosslinking agent with a higher average molecular weight for the synthesis of polymer-based hydrogel materials (i.e., PVP with an average molecular weight of 58,000 g/mol) is likely most advantageous in terms of resistance to stress. Thus, the simultaneous use of PEGDA 700 and PVP with the abovementioned average molecular weight resulted in the formation of a polymer network with promising resistance to external forces.

Considering the results presented in Figure 2a, the impact of the amount of PEGDA 575 in the hydrogel on its percentage elongation was negligible. Therefore, photopolymerization proceeded to a similar degree in samples PEGDA 575—2.0 and PEGDA 575—2.5, and the percentage elongation of both samples was approximately 53%. In the case of samples PEGDA 700—1.5, PEGDA 700—2.0, and PEGDA 700—2.5, the more crosslinking agent in hydrogel sample, the lower its elongation. This result is probably related to the relaxation of polymer chains. The simultaneous use of a crosslinking agent with a high average molecular weight (i.e., PEGDA 700) and PVP with an average molecular weight of 58,000 g/mol resulted in the formation of densely crosslinked network of polymer chains. Therefore, the polymer chains had insufficient space to stretch, resulting in a low percentage elongation of the material.

### 2.2. Results of Incubation Studies

**Figure 3:**
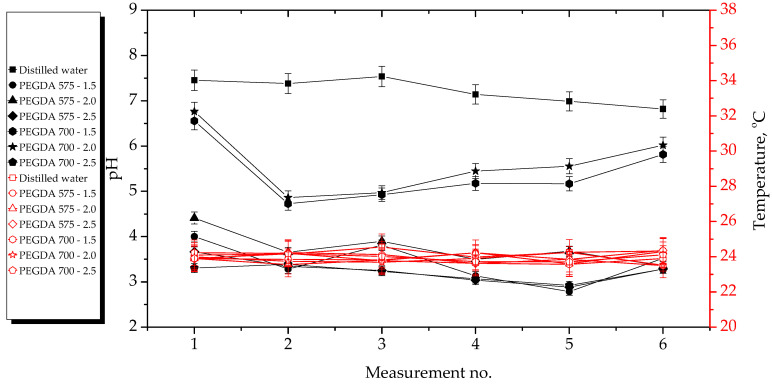
Results of hydrogel incubation in distilled water.

**Figure 4:**
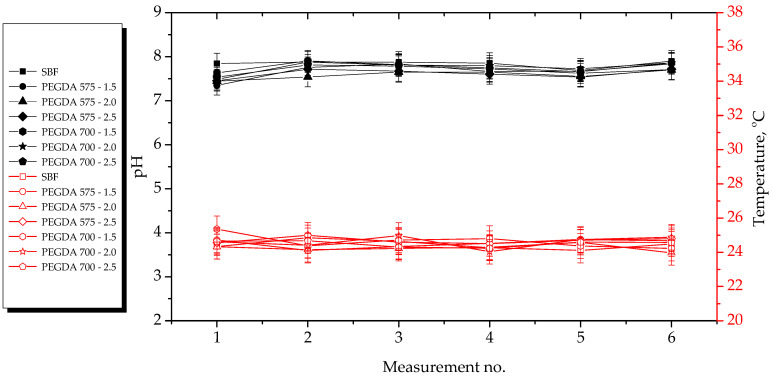
Results of hydrogel incubation in SBF.

**Figure 5:**
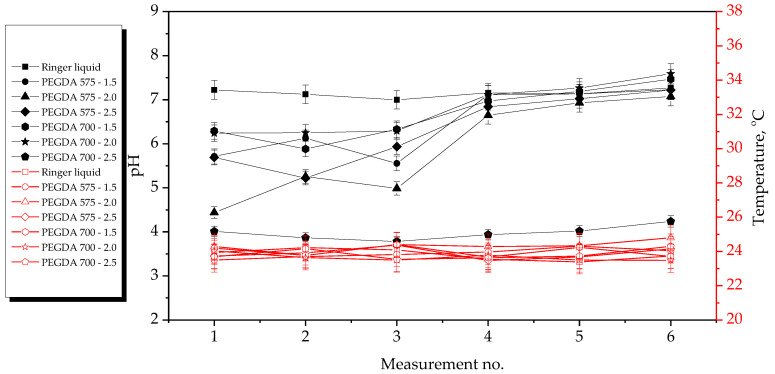
Results of hydrogel incubation in Ringer solution.

**Figure 6:**
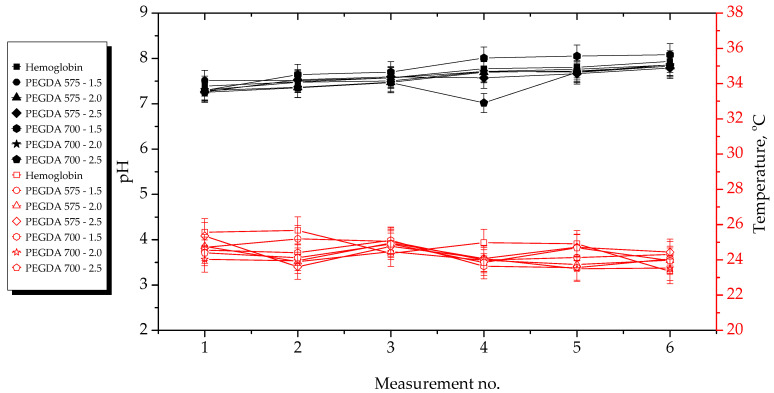
Results of hydrogel incubation in hemoglobin.

The results of the incubation studies, including the temperature and pH values of particular liquids measured during hydrogel incubation, are presented in Figure 3, Figure 4, Figure 5 and Figure 6. All studies were performed in triplicate for each sample, and the results are presented as mean value ± standard deviation (SD).

According to the results presented above, the hydrogels showed the highest stability in SBF and 2% hemoglobin solution because the pH values of the liquids only changed to a limited extent during the incubation period. Such insignificant changes may be explained by the dense crosslinking of the hydrogel networks that likely occurred during their incubation both in SBF, which contains numerous divalent ions, and in protein-containing hemoglobin solution.

On the other hand, the most considerable changes in pH values were observed in the case of distilled water, which contains neither ions nor proteins that could interact with the hydrogel network, thus forming additional crosslinks and limiting the release of the modifying agents (*Aloe vera* juice and vitamin C). Both these modifiers are acidic; thus, when they were released from the hydrogel matrix as a result of loosening of the polymer chains, the pH value of the distilled water decreased from approximately 7.5 to approximately 3.0. In the case of Ringer solution, the changes in its pH demonstrated the buffering properties of the hydrogels. This incubation medium contains fewer divalent ions (such as Ca^2+^) than SBF; thus, the modifiers may have been released. Notably, the pH value of this Ringer solution changed until it was stabilized with at the fourth measurement.

Importantly, the lack of significant differences in pH values between the tested samples proved that the incubation medium had a greater impact on the release of the modifying agent than the amount or the average molecular weight of the crosslinking agent.

Incubation studies were also performed to verify whether the tested hydrogels degraded in the tested environments. Degradation of such materials could lead to a permanent decrease or increase in the pH of the incubation medium as a result of the formation of degradation products. The lack of increase in pH values showed that the analyzed materials did not degrade in incubation environments. FT-IR spectra of the hydrogels before and after incubation were compared to verify this conclusions. The results of spectroscopic analysis are presented in next subsection.

### 2.3. Analysis of Hydrogel Structure Using FT-IR Spectroscopy

**Figure 7:**
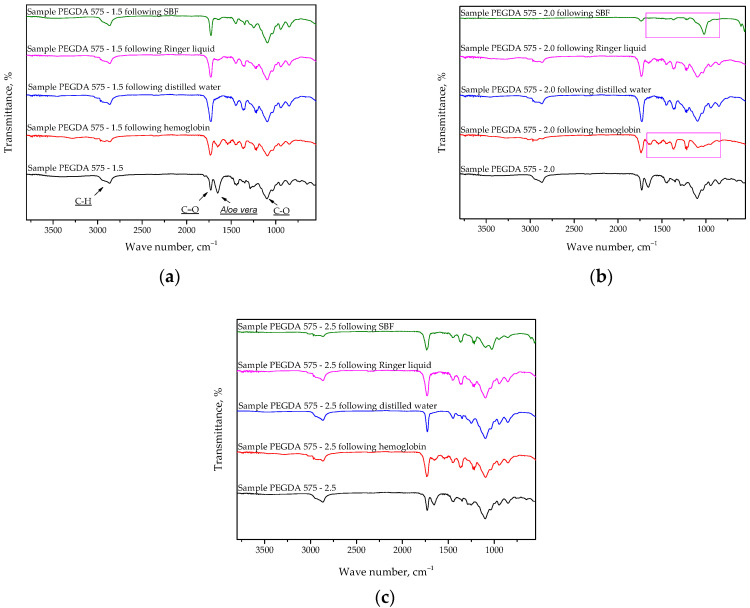
FT-IR spectra showing the influence of incubation on the structure of hydrogels obtained using varying amounts of PEGDA 575, i.e., 1.5 mL (**a**), 2.0 mL (**b**) and 2.5 mL (**c**).

**Figure 8:**
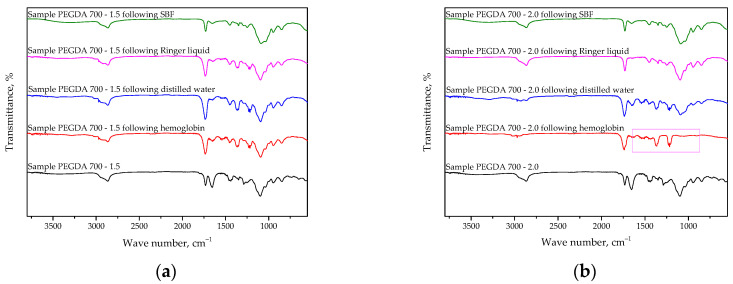

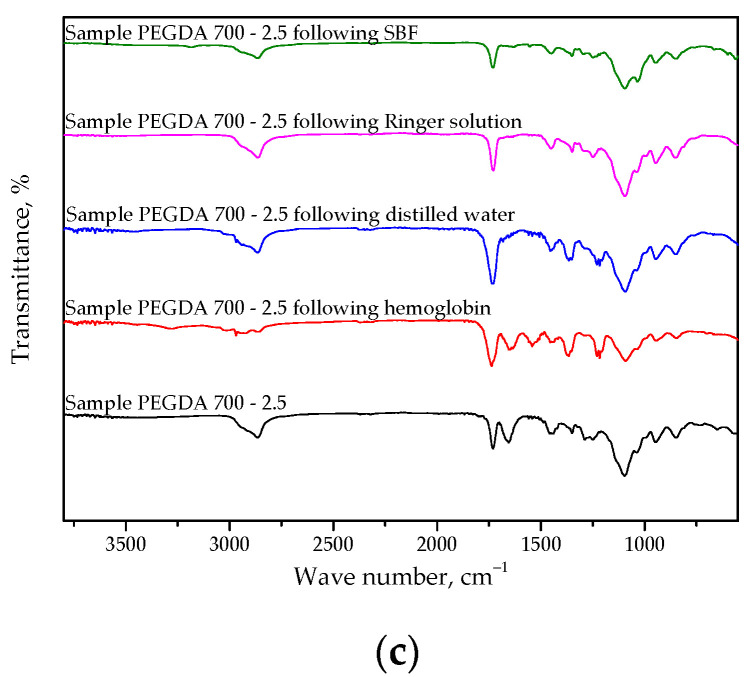
FT-IR spectra showing the influence of incubation on the structure of hydrogels obtained using varying amounts of PEGDA 700, i.e., 1.5 mL (**a**), 2.0 mL (**b**) and 2.5 mL (**c**).

FT-IR spectroscopy results are presented in Figure 7 and Figure 8. The FT-IR spectra of hydrogels before and after incubation in simulated physiological liquids were summarized to verify the impact of the study on their structure.

FT-IR analysis was used to determine the potential changes in the structure of hydrogels obtained using varying amounts of crosslinking agent with an average molecular weight of both 575 g/mol and 700 g/mol. Table 1 shows the absorption bands observed in the spectra with corresponding chemical bonds, as well as the type of vibration.

The lack of differences in the intensities of particular absorption bands in the FT-IR spectra of most the of tested samples indicates that the samples did not degrade during incubation. A significant reduction in the intensities of all absorption bands visible in the obtained FT-IR spectra would have proven degradation of the hydrogels; however, no such changes were observed. A decrease in the intensity of some absorption bands or their total disappearance (indicated by pink rectangles in the spectra) was only observed in the case of the FT-IR spectra of samples PEGDA 575—2.0 and PEGDA 700—2.0, which were incubated in SBF and 2% hemoglobin solution. However, FT-IR spectroscopy constitutes a point analysis, so some vibration characteristic, e.g., for modifiers, might be obscured by other vibrations. Additionally, the absorption band characteristic of polysaccharides included in *Aloe vera* juice disappeared, which may indicate the release of this modifier from the hydrogel matrix. Such a phenomenon was also discussed with respect to the incubation studies, wherein the observed decrease in the pH of distilled water may have also been caused by the release of this substance.

### 2.4. Sorption Properties of Hydrogels

**Figure 9:**
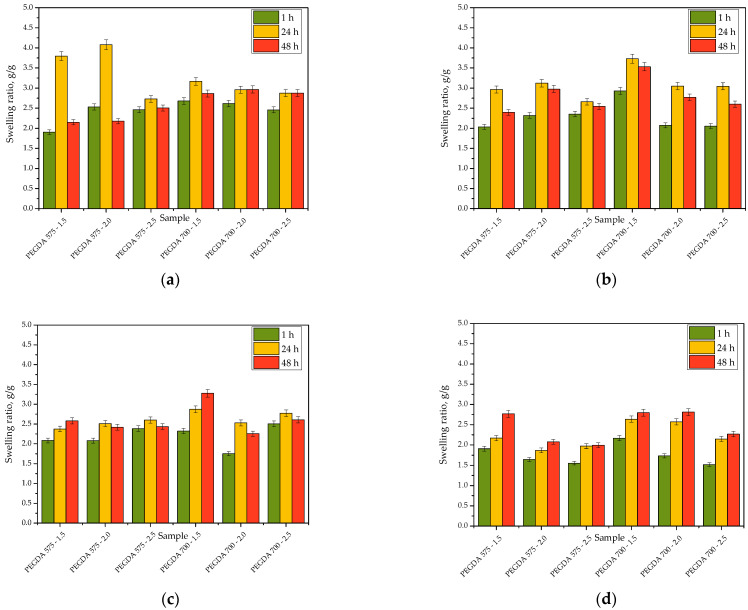
Swelling ability of hydrogels in distilled water (**a**), SBF (**b**), Ringer solution (**c**), and hemoglobin (**d**).

The sorption properties of the hydrogels were verified in simulated physiological liquids and in distilled water as a reference liquid. The swelling ratios calculaetd for each hydrogel sample in each tested medium are presented in Figure 9. The study was performed in triplicate for each sample, and the results are presented as mean ± standard deviation (SD).

All hydrogels exhibited sorption capacity in all tested liquids, with swelling ratios within the range of 1.5 g/g–4.0 g/g. The highest swelling ratio was calculated for sample PEGDA 575—1.5 in distilled water, probably because, in contrast to the other tested liquids, distilled water contains neither divalent ions nor proteins that could interact with the polymer chains of the hydrogel network, thus increasing its crosslinking density. Considering the swelling ratios of samples PEGDA 575—2.0, PEGDA 575—2.5, PEGDA 700—1.5, PEGDA 700—2.0, and PEGDA 700—2.5, the more crosslinker included in the hydrogel, the lower its swelling ability. An increase in the amount of PEGDA in the hydrogel matrix leads to an increase in its crosslinking density, limiting the penetration of the absorbed liquid into the spaces between the polymer chains, as similarly reported by Hoti et al. [33] and Khan and Ranjha [34].

The most considerable differences in the swelling ratios for the same hydrogels tested in various liquids were observed in the case of samples PEGDA 575—1.5 and PEGDA 575—2.0 (materials with the lowest crosslinking density). These samples showed significantly lower ability to absorb SBF, Ringer solution, and 2% hemoglobin solution relative to their ability to absorb distilled water, which is likely due to the presence of divalent calcium ions both in SBF and Ringer solution, which, as previously mentioned, may interact with the polymer network, increasing its crosslinking degree. Similar results with respect to the impact of these ions on the swelling ability of hydrogels was also reported by Bai et al. [35].

In most cases, the hydrogels swelled to the least extent in 2% hemoglobin solution, which may be explained by the presence of proteins in this medium, which could have contributed to the highest crosslinking degree of the hydrogel network among all tested media, leading to low swelling ratios.

Considering the results obtained for samples PEGDA 575—2.5 and PEGDA 700—2.5 (i.e., the samples with the highest amount of PEGDA 575 and PEGDA 700) their swelling ratios in all tested liquids do not differ considerably from each other. These hydrogels showed a high crosslinking density, and neither calcium ions (included in SBF and Ringer solution) nor proteins (included in 2% hemoglobin solution) affected this parameter. As a result, the sorption capacity of these materials in SBF, Ringer solution, and 2% hemoglobin solution was similar to their swelling ability determined in distilled water.

The high swelling ratio of all tested hydrogel samples was observed just after the 1 h. In the case of SBF and distilled water, the highest swelling ratios were determined after 24 h, indicating that the material absorbed the maximum possible amount of liquid. In turn, after 48 h, the swelling ratios in these liquids decreased, probably due to the release of the modifying agents, i.e., *Aloe vera* juice and vitamin C, from the hydrogel matrices. On the other hand, this phenomenon could also be explained by the degradation of the analyzed samples. The results of both the incubation studies and FT-IR analysis discussed previously suggest that the mentioned substances were likely released, as indicated by the significant decrease in the swelling ratio after 48 h, the decrease in pH values of distilled water during hydrogel incubation, and the decrease in the intensity of the absorption band corresponding to the polysaccharides included in *Aloe vera* juice. Similar results were not observed in the case of samples tested in Ringer solution and 2% hemoglobin solution; the swelling ratios in these liquids increased with time, indicating that the maximum sorption of these samples was not achieved within 48 h.

### 2.5. Characterization of Hydrogels via Microscopic Techniques

**Figure 10:**
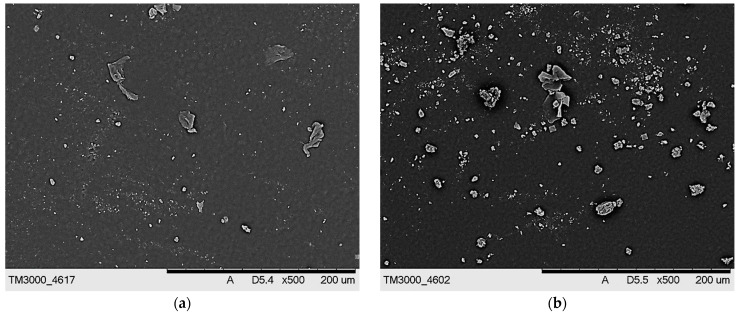

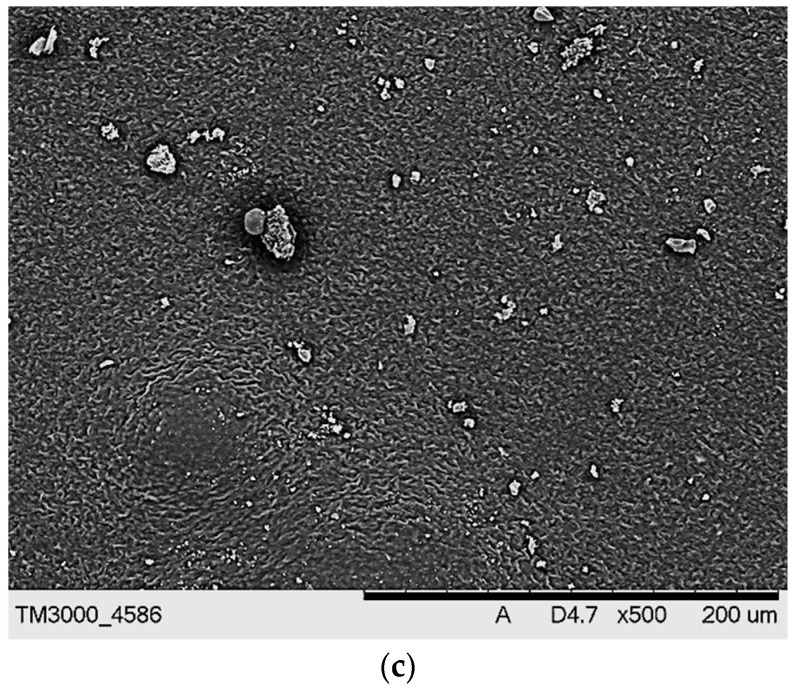
SEM images of hydrogel samples containing PEGDA 575: (**a**) 1.5 mL, (**b**) 2.0 mL, and (**c**) 2.5 mL.

**Figure 11:**
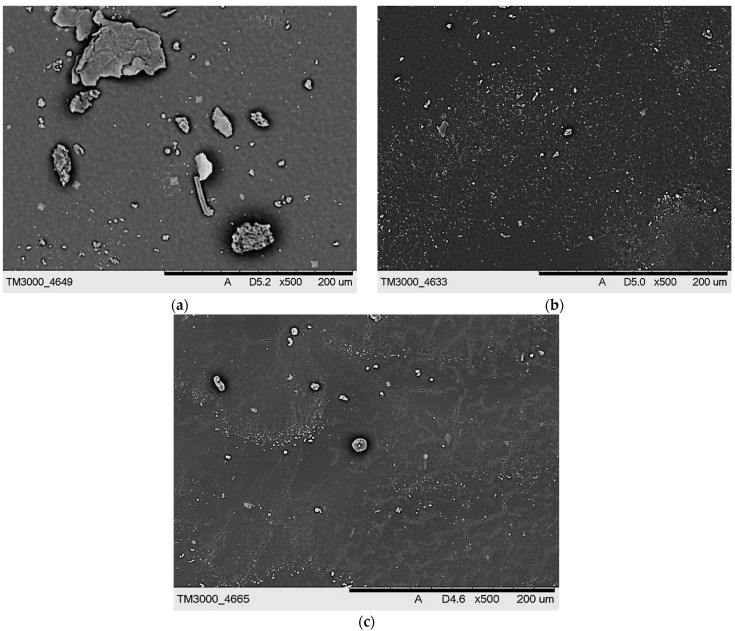
SEM images of hydrogel samples containing PEGDA 700: (**a**) 1.5 mL, (**b**) 2.0 mL, and (**c**) 2.5 mL.

SEM images of hydrogels presenting their surface morphology are shown in Figure 10 and Figure 11.

The obtained SEM images show the lack of significant differences between the surface morphology of dry hydrogels. It was assumed that the type of crosslinking agent used during the photopolymerization process or its concentration in the reaction mixture would significantly affect the surface morphology of the hydrogels. However, such differences were not observed, probably due to the presence of the modifying agents (*Aloe vera* juice and vitamin C) in the hydrogel matrices, which filled out the outer pores of the hydrogels, thus smoothing their surface. Such a smoothing of the hydrogel surfaces caused by the presence of additives in their matrices was also described by Rahman et al. [36].

**Figure 12:**
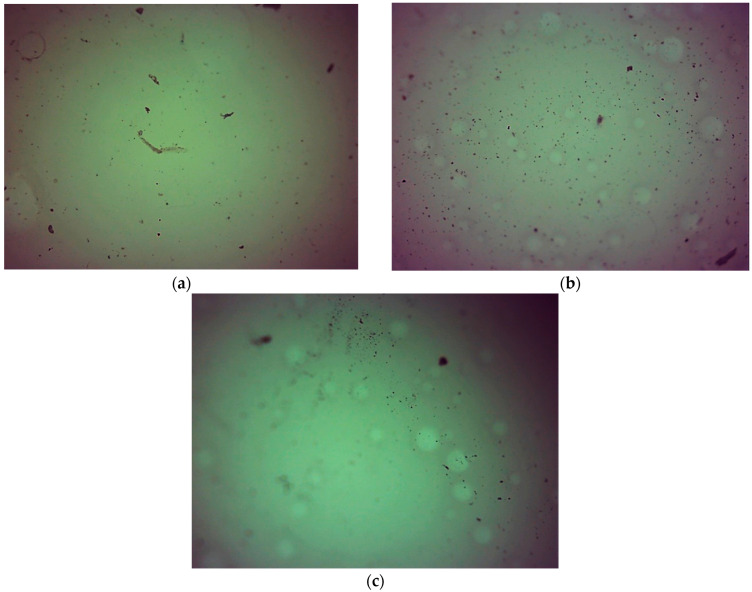
Optical images of hydrogels obtained using PEGDA 575: (**a**) 1.5 mL, (**b**) 2.0 mL, and (**c**) 2.5 mL (magnification 40×).

**Figure 13:**
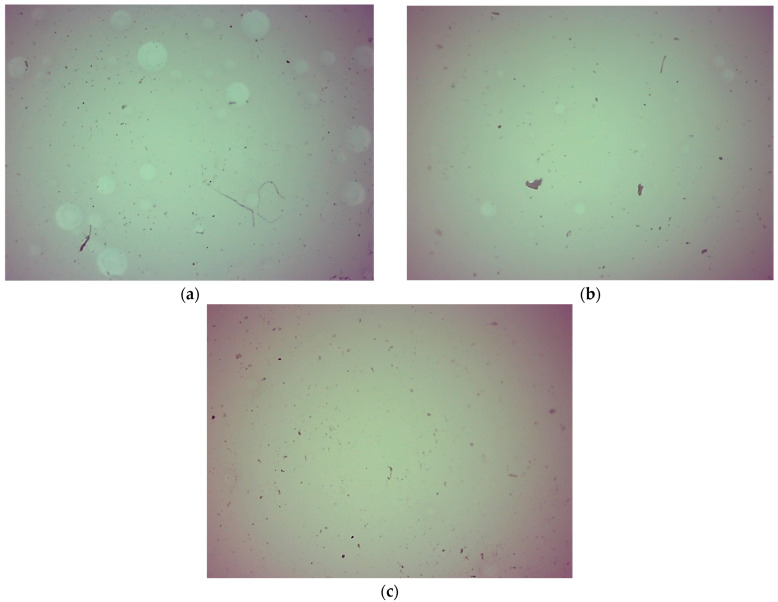
Optical images of hydrogels obtained using PEGDA 700: (**a**) 1.5 mL, (**b**) 2.0 mL, and (**c**) 2.5 mL (magnification 40×).

Figure 12 and Figure 13 show images of hydrogels obtained using via optical microscopy.

No differences in surface morphology were observed in optical images of hydrogels with varying content of crosslinking agent with an average molecular weight of 575 g/mol or 700 g/mol. We also assumed that it would be possible to notice differences between samples obtained using varying amount of crosslinking agent or crosslinker with varying average molecular weights. The lack of differences may have resulted from the presence of modifiers in the hydrogel matrices, which filled up the hydrogels’ pores. These observations and conclusions are consistent with SEM imaging results.

## 3. Materials and Methods

### 3.1. Materials

*Aloe vera* juice acting as an active substance and introduced into the hydrogel matrix was purchased from Herbal Pharmaceuticals (Krakow, Poland). All other reagents, i.e., vitamin C (an active substance, L-ascorbic acid, an ACS reagent; ≥99%), polyvinylpyrrolidone (the basic component of the polymer network, PVP; average molecular weight: 58,000 g/mol), diacrylate poly(ethylene glycol) (used as a crosslinker, PEGDA; average molecular weight: 575 g/mol and 700 g/mol), and 2-hydroxy-2-methylpropiophenone (Darocur^®^ 1173, 97%; used to initiate the photopolymerization process), were purchased from Sigma Aldrich (Saint Louis, MO, USA). All reagents were used without further purification.

### 3.2. Synthesis of Hydrogel Dressings via UV-Induced Polymerization

Hydrogels were prepared via a polymerization process induced by UV radiation. To this end, reaction mixtures consisting of adequate amounts of all reagents (i.e., PVP solution, PEGDA, modifiers (*Aloe vera* juice and vitamin C solution), and photoinitiator) were mixed thoroughly and placed near an EMITA VP-60 UV lamp (λ = 320 nm; power: 180 W; manufacturer: Famed, Lodz, Poland) for 120 s. Detailed compositions of all prepared hydrogels samples are listed in Table 2.

**Figure 14:**
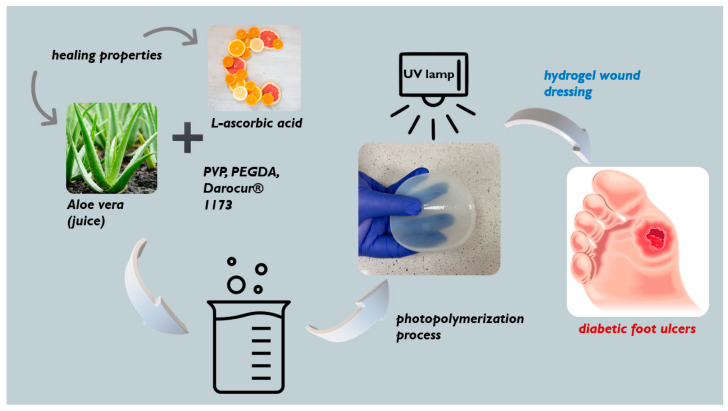
Preparation protocol for the hydrogel dressings.

The process of the preparation of the hydrogel dressings schematically represented in Figure 14.

The hydrogels were then dried at 37 °C and stored for experiments to determine their sorption properties, surface morphology, and selected mechanical characteristics.

### 3.3. Investigations on the Elongation and Tensile Strength of Hydrogels

The first studies involved determining elongation and tensile strength of the prepared hydrogels. Analyses were performed at ambient temperature with a universal testing machine (Shimadzu, Kyoto, Japan). Paddle-shaped hydrogel samples (cut using a ZCP020 manual blanking press) were fixed within the jaws of the testing machine, and the experiment was carried out until the samples cracked. The procedure allowed us to verify the mentioned parameters, i.e., the percentage elongation (A), which was calculated using Equation (1); and the tensile strength, which was calculated using Equation (2); both equations are provided below:

(1) $$A=\frac{I_{u}-I_{0}}{I_{0}}\times 100\%$$

(2) $$R_{m}=\frac{F_{m}}{S_{0}}$$

where A is the percentage elongation, $I_{u}$ is the measured length after the hydrogel was broken, $I_{0}$ is the measured length of the hydrogel sample before the experiment, $R_{m}$ is the tensile strength, $F_{m}$ is the maximum strength, and $S_{0}$ is the cross-sectional area of analyzed material before the experiment.

### 3.4. Incubation Studies of Hydrogels in Selected Simulated Physiological Liquids

As part of these experiments, hydrogel samples (with a weight of approximately 1.0 g) were immersed for a specific period in 50 mL of selected liquids simulating the environments of human organisms, i.e., simulated body fluid (SBF), Ringer solution, and hemoglobin (2% solution). The study was also performed in distilled water as a reference liquid. Incubation was conducted at 37 °C to simulate conditions of the human body. During hydrogel immersion, pH values and the temperature of incubation media were regularly measured using a multifunctional ELMETRON CX-701 m (Elmetron, Zabrze, Poland).

### 3.5. Analysis of Hydrogels Using Fourier Transform Infrared (FT-IR) Spectroscopy

The presence of specific functional groups in the hydrogel structures was verified via FT-IR spectroscopy. The study was conducted at ambient temperature using the following equipment: a Thermo Scientific Nicolet iS5 FT-IR spectrophotometer (Thermo Fisher Scientific, Waltham, MA, USA). The spectra were recorded within the range of 4000–500 cm^−1^ using 32 running scans at a resolution of 4.0 cm^−1^.

### 3.6. Sorption Properties of Hydrogels

The sorption properties of the hydrogels were evaluated using the same liquids used in the incubation studies. The study consisted of placing a dry hydrogel sample (weight: approximately 1.0 g) in a selected liquid for specific period (1 h, 24 h, and 48 h), separating it from the solution, and weighing again. The sorption properties of the tested samples were characterized using swelling ratios (*α*), which were calculated by means of Equation (3):

(3) $$\alpha =\frac{\left(m-m_{0}\right)}{m_{0}}$$

where *α* is the swelling ratio, g/g; *m* is the mass of the swollen hydrogel (g), and *m*_0_ is the mass of the dry hydrogel (g).

### 3.7. Microscopic Analysis of Hydrogels

Before all microscopic analyses, the hydrogels were dried for 24 h at 37 °C. First, the surface morphology of developed materials was characterized by a Jeol 5510LV scanning electron microscope (Jeol Ltd., Tokyo, Japan). Importantly, before the analyses, dry hydrogels were sputtered with gold to provide adequate conductivity.

Finally, a Delta Optical Genetic Bino optical microscope (Delta Optical, Warszawa, Poland) was used to obtain optical images of the hydrogels.

Both microscopic analyses were performed to characterize the surface morphology of the obtained hydrogels. Before measurements we taken, the samples were dried at 37 °C for 24 h.

## 4. Conclusions

- Both the amount of crosslinking agent used during the photopolymerization process and its average molecular weight affected both the tensile strength and percentage elongation of the hydrogels. The more crosslinking agent in the hydrogel matrix, the higher its tensile strength, which was related to the increase in the crosslinking density of the hydrogels. On the other hand, the more crosslinking agent in the hydrogel sample, the less elongation.
- Hydrogels showed the highest stability during incubation in SBF and 2% hemoglobin solution. The most considerable changes in pH values during hydrogel incubation were observed in the case of distilled water. The significant observed pH decrease was probably due to the release of *Aloe vera* juice from the hydrogel matrices to the incubation medium. In the case of Ringer solution, the changes in pH demonstrated the buffering properties of the hydrogels.
- The absorption band characteristic of the polysaccharides included in *Aloe vera* juice was disappeared from the FT-IR spectra of the hydrogels after incubation, possibly indicating the release of this modifier from the hydrogel matrix and confirming our assumptions about the reason for the pH decrease in distilled water.
- All hydrogels showed swelling ability. The highest sorption capacity was observed in distilled water due to the lack of divalent ions or proteins that could have increase the crosslinking degree of the hydrogels, thus decreasing their swelling ability, as observed in the case of other tested solutions. The higher the content of the crosslinking agent in the hydrogel, the lower its swelling ability.
- Hydrogels showed similar surface morphology, irrespective of the content of the crosslinking agent and its average molecular weight, probably due to the presence of modifiers (*Aloe vera* juice and vitamin C) in hydrogel matrices, which filled up the hydrogels’ pores and made their surfaces smooth.

## Figures and Tables

**Table 1:** Absorption bands in FT-IR spectra with corresponding chemical bonds, as the type of vibrations.

Wavenumber, cm^−1^	Chemical Bond	Type of Vibration
2850	C-H	Stretching
1750	C=O	Stretching
1100	C-O	Stretching

**Table 2:** Compositions of the hydrogels.

Sample *	15% PVP Solution, mL	*Aloe vera* Juice, mL	5% Vitamin C Solution, mL	PEGDA 575, mL	PEGDA 700, mL	Photoinitiator, mL
PEGDA 575—1.5	7.0	3.0	2.0	1.5	-	0.25
PEGDA 575—2.0				2.0		
PEGDA 575—2.5				2.5		
PEGDA 700—1.5				-	1.5	
PEGDA 700—2.0					2.0	
PEGDA 700—2.5					2.5	

* Sample names reflect the name of the crosslinking agent, with number referring to their average molecular weight and the amount used during sample synthesis.

## Data Availability

Data sharing is not applicable for this article.
